# Digital Data Collection Reduces Terminal Digit Bias in Haemodynamic and Physical Activity Measures in Pulmonary Arterial Hypertension

**DOI:** 10.1002/pul2.70290

**Published:** 2026-04-03

**Authors:** Alexander M. K. Rothman, Marcelle Paula‐Ribeiro, Eleanor McNally, James Wason, Mark Toshner

**Affiliations:** ^1^ Division of Clinical Medicine University of Sheffield Sheffield UK; ^2^ Sheffield Pulmonary Vascular Disease Unit, Royal Hallamshire Hospital, Sheffield Teaching Hospitals NHS Foundation Trust UK; ^3^ Population Health Sciences Institute Newcastle University UK; ^4^ Victor Phillip Dahdaleh Heart and Lung Research Institute University of Cambridge UK; ^5^ Royal Papworth Hospital, Papworth Rd, Trumpington Cambridge UK

**Keywords:** bias, haemodynamics, Pulmonary arterial hypertension, right heart catheterisation

## Abstract

Accurate measurement of cardiopulmonary hemodynamics and exercise capacity is vital for PAH diagnosis and management. Terminal digit bias in clinical measures introduces non‐differential error. Remote monitoring using implanted devices eliminated this bias, demonstrating its potential to enhance accuracy in both clinical practice and research by reducing variability in observer‐reported data.

## 1 Introduction

Pulmonary arterial hypertension (PAH) is a rare progressive disease with significant morbidity. Randomised clinical trials of PAH therapies use endpoints including cardiac output, pulmonary arterial pressure, and 6‐min walk distance [1]. Terminal digit preference describes a source of bias, where the final digit of a number is rounded by the observer, typically to 0 and 5, which leads to over‐representation in measurement readings. In phase III clinical trials of PAH therapies, terminal digit preference in values of pulmonary artery pressure and 6‐min walk distance has been demonstrated [2]. This bias has the potential to introduce inaccuracies at the point of diagnosis and in the assessment of the efficacy of therapeutic interventions. Technological advances provide the capacity to remotely measure cardiopulmonary haemodynamics and physical activity, daily from a patient's home using minimally invasive, implanted devices [3, 4]. We evaluated whether terminal digit preference affected remote measurements of heart rate, physical activity, cardiac output, and pulmonary artery pressure in patients with pulmonary arterial hypertension with implanted devices.

## 2 Methods

Following enrolment in Feasibility of Novel Clinical Trial Infrastructure, Design and Technology for Early Phase Studies in Patients with Pulmonary Hypertension (FIT‐PH, approved by the Yorkshire & The Humber research ethics committee: 19/YH/0354), patients underwent standard clinical evaluation followed by right heart catheterisation, and implantation of a pulmonary artery pressure (PAP) monitor (CardioMEMS, Abbott) and insertable cardiac monitor (LinQ, Medtronic) [5, 6]. Daily, remote measures of heart rate, physical activity (measured as minutes of activities of daily living), cardiac output, and PAP were relayed to the clinical care team through secure online portals. Assuming a uniform distribution of terminal digits, each digit (0–9) should be represented at 10%. Deviation from this distribution was tested for systolic, diastolic, and mean PAP, cardiac output and physical activity using a *χ* ^2^ test. Analyses used SPSS version 29 for MacOS (IBM).

## 3 Results

**Figure 1:**
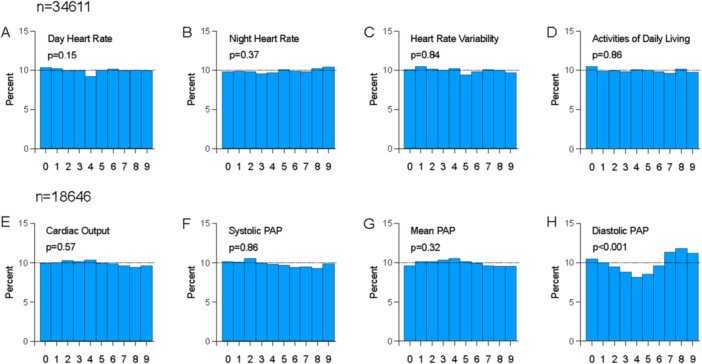
Distribution of terminal digits in remote monitoring data for (A) day heart rate, (B) night heart rate, (C) heart rate variability, (D) activities of daily living, (E) cardiac output, (F) systolic pulmonary artery pressure, (G) mean pulmonary artery pressure, and (H) diastolic pulmonary artery pressure. The percentage of measurements with each terminal digit is represented on the y‐axis, and the terminal digit on the x‐axis. *P*‐values are from chi‐square tests. *n* = 34,611 insertable cardiac monitors and *n* = 18,646 pulmonary artery pressure monitors, derived measurements (follow‐up) for PA pressures.

Thirty‐five patients underwent right heart catheterisation with implantation of minimally invasive remote monitoring devices (Table 1). There were no device‐related serious adverse events and two device‐related adverse events (one minor haemoptysis and one insertable cardiac monitor wound dehiscence at day 3) [5]. Following implantation, 18,646 independent measures of PAP and cardiac output and 34,611 independent measures of heart rate, heart rate variability and physical activity were made between November 2019 and May 2023. The distribution of terminal digits for systolic and mean pulmonary artery pressure, and cardiac output and physical activity did not deviate from the expected representation (Figure 1A–G). The terminal digits for measures of diastolic PAP were not equally represented (Figure 1H). The digits 7, 8 and 9 were significantly overrepresented.

**Table 1:** Baseline patient characteristics.

Demographics	*N* = 35
Age (years, mean ± SD)	51 (28.5)
Gender (*n*,% female)	25 (71.4)
Ethnicity (*n*, %)	
White	33 (94.3)
Asian	2 (5.7)
BMI (mean ± SD)	27.9 (5.7)
6MWDT (mean ± SD)	362.9 (231.7)
WHO Functional Class (*n*, %)	
Class I	0
Class II	1 (2.9)
Class III	29 (82.9)
Class IV	0///5 (14.3)
NT‐proBNP (mean ± SD)	666.2 (1065.5)
PH sub‐diagnosis (*n*, %)	
IPAH	28 [80]
HPAH	1 (2.9)
CTD	4 (11.4)
PVOD	1 (2.9)
CHD	1 (2.9)
Cardiopulmonary Haemodynamics (mean ± SD)	
CI	2.8 (0.7)
mPAP	46.2 (13.5)
PVR	615 (358)
mRAP	8.6 (4.6)

*Note:* Data are presented as numbers (%) for categorical variables, mean (standard deviation) for normally distributed continuous variables, and median (interquartile range) where appropriate.

Abbreviations: 6MWDT, 6‐minute walk test; BMI, body mass index; CHD, congenital heart disease; CI, cardiac index; CTD, connective tissue disease; HPAH, heritable pulmonary arterial hypertension; IPAH, idiopathic pulmonary arterial hypertension; mRAP, mean right atrial pressure; mPAP, mean pulmonary artery pressure; NT‐proBNP, N‐terminal pro‐B‐type natriuretic peptide; PVOD, pulmonary veno‐occlusive disease; PVR, pulmonary vascular resistance; WHO, World Health Organisation.

## 4 Discussion

Terminal digit preference is most frequently a function of observer bias and has been described in studies of patients with systemic hypertension, where it is associated with lower mean pressure and higher numbers of cardiovascular events [7]. Accurate measures of PAP, cardiac output and exercise capacity (most commonly 6‐min walk distance) are critical in the diagnosis and management of patients with pulmonary arterial hypertension, and the evaluation of therapeutic response in clinical studies [1, 8]. However, in phase III randomised clinical trials of PAH therapies, terminal digits 0 and 5 are overrepresented in measures of PAP and 6‐min walk distance [2]. In such clinical studies, patients undergo right heart catheterisation before and after administration of drug, meaning that rounding of values based on terminal digit preference introduces measurement error on two occasions. This non‐differential error biases studies towards a null outcome. In clinical practice, right heart catheterisation is used for clinical diagnosis and evaluation of treatment response [1]. In this context, terminal digit preference may alter which patients meet diagnostic criteria and influence ongoing clinical management decisions [8, 9, 10]. As such, methods that provide accurate, unbiased measures of PAP and cardiac output offer potential advantages. In clinical studies, reduced error may increase study power and reduce the number of patients exposed to drugs and the study duration [3]. In clinical practice, accurate haemodynamic evaluation may ensure clinical decisions are made based on accurate measures. In both circumstances, the use of computer‐generated measures and/or core‐lab analysis may be preferable to physician‐interpreted measures, which are subject to terminal digit preference.

Technological advances have provided the capacity to remotely measure cardiopulmonary haemodynamics and physical activity from the patient's home. Digital measures of PAP and physical activity are measured as integers and cardiac output as decimals. In contrast to operator‐reported values in phase III studies, the present study demonstrates that terminal digit preference was not present in systolic and mean pulmonary artery pressure, cardiac output, heart rate, heart rate variability and physical activity captured using remote, digital technology. A terminal digit preference was observed for 0, 7, 8 and 9 in remote, digitally reported diastolic PAP Assuming a uniform distribution of terminal digits should be represented at 10%. The normal range for diastolic PAP is 8‐12 mmHg, and values of less than 5 mmHg are uncommon [11]. As such, there is a cut‐off at the lower physiological range of diastolic PAP, which biases the distribution of values, meaning the assumption of uniform distribution of terminal digits is not valid for this value.

## 5 Conclusions

We found that digital data collection prevents the described observer‐mediated preference for a terminal digit of 0 or 5 in measurements of systolic and mean PAP in patients with pulmonary arterial hypertension. The minimisation or removal of terminal digit preference bias has important implications for research and clinical care in pulmonary hypertension, in which measures of PAP, cardiac output, exercise capacity and physical activity are increasingly used for the evaluation of therapeutic efficacy.

## Author Contributions

The study was conceived by Mark Toshner and Alexander M. K. Rothman. All authors contributed to the acquisition, analysis and interpretation of the data, revised the manuscript and approved the submitted version of the manuscript. Alexander M. K. Rothman is guarantor of the data.

## Ethics Statement

All patients provided informed consent. Feasibility of Novel Clinical Trial Infrastructure, Design and Technology for Early Phase Studies in Patients with Pulmonary Hypertension was approved by the Yorkshire & The Humber research ethics committee: 19/YH/0354.

## Conflicts of Interest

AMKR has received grants and research support from: MRC Experimental Medicine Award (MR/W026279/1), British Heart Foundation (FS/CRTF/23/24465, HI/B3/24/370100), EPSRC Project Grant (EP/Z536027/1, EP/Z531297/1), Innovate UK (TS/Y02477X/1), Novartis, JAbbott, Medtronic, MSD, Gradient, Relief Cardiovascular, Jupiter. MT has received support from NIHR Cambridge BRC, MRC Development Funding Pathway Scheme Award (MR/T025646/1), Jansen, Apollo, Merk, RxPx, uMed, GSK, ComCov, FluCov.
